# Human genome editing in clinical applications: Japanese lay and expert attitudes

**DOI:** 10.3389/fgene.2023.1205092

**Published:** 2023-08-17

**Authors:** Tsutomu Sawai, Taichi Hatta, Kyoko Akatsuka, Misao Fujita

**Affiliations:** ^1^ Graduate School of Humanities and Social Sciences, Hiroshima University, Higashi-Hiroshima, Japan; ^2^ Institute for the Advanced Study of Human Biology (ASHBi), Kyoto University, Kyoto, Japan; ^3^ Shizuoka Graduate University of Public Health, Shizuoka, Japan; ^4^ Uehiro Research Division for iPS Cell Ethics, Center for iPS Cell Research and Application, Kyoto University, Kyoto, Japan

**Keywords:** heritable genome editing, somatic cell genome editing, survey, public attitudes, research ethics, Japan

## Abstract

**Background:** The world’s first gene-edited babies, reported by the Chinese scientist He Jiankui, prompted an outcry of criticism and concerns worldwide over the use of genome editing for reproductive purposes. Many countries and academic associations opposed to heritable genome editing (HGE) called for public discussion involving various stakeholders. To hold a discussion of this nature and form a consensus concerning HGE, we must understand under what conditions stakeholders consider HGE acceptable and the reasons for which they deem it unacceptable.

**Methods:** Laypeople and researchers were surveyed in May 2019. They were asked about the degree of their acceptance toward somatic genome editing (SGE) and HGE; those who answered “acceptable depending on the purpose” were queried further regarding their acceptance in the contexts of specific clinical purposes.

**Results:** Responses were obtained from 4,424 laypeople and 98 researchers. The percentage of respondents choosing each option in attitudes to HGE was, from largest to smallest: “acceptable depending on purpose” (laypeople 49.3%; researchers 56.1%), “not acceptable for any purpose” (laypeople 45.8%; researchers 40.8%), and “acceptable for any purpose” (laypeople 5.0%; researchers 3.1%). In an additional question for those who answered “acceptable depending on the purpose,” laypeople found the following purposes acceptable: infertility treatment (54.5%), treatment of life-threatening diseases (52.2%), and treatment of debilitating diseases (51.4%). Meanwhile, the degree of acceptance for enhancement purposes was 10.7, 7.9, 6.2, and 5.5% for physical, cognitive, health, and personality enhancements, respectively. In contrast, acceptance among the researchers was 94.5% and 92.7% for the treatment of life-threatening and debilitating diseases, respectively, compared with 69.1% for infertility treatment. Researchers’ acceptance for enhancement purposes was similar to that of the lay participants, with 12.7, 9.1, 10.9, and 5.5% for physical, cognitive, health, and personality enhancement, respectively.

**Conclusion:** In the past, debates regarding the acceptability of human genome editing in clinical applications tend to focus on HGE in many countries. Society will now need to debate the acceptability of both types of human genome editing, HGE and SGE.

## 1 Introduction

CRISPR-Cas9, a genome editing technology that emerged in 2012, allows scientists to modify genes more efficiently and accurately than previous technologies. It was used in April 2015 by scientists who, for research purposes, performed genome editing in human embryos for the first time (Liang et al., 2015). Since then, the moral acceptability of genome editing for reproductive purposes has been debated (e.g., NASEM, 2015; NCB, 2016). In clinical genome editing in the germline, which includes germ cells and embryos, the results of genetic modifications to an individual can be passed on to their children as well as to subsequent generations, unlike somatic genome editing (SGE); therefore, several countries and academic associations believe that heritable genome editing (HGE) should be banned (Brokowski, 2018). In November 2018, Chinese scientist He Jiankui reported the birth of twin girls whose genomes had been edited at the embryonic stage to prevent parent-to-child transmission of the human immunodeficiency virus (HIV) (Regalado, 2018). This report prompted an outcry of criticism and concerns worldwide over using genome editing for reproductive purposes because safety and ethical issues have not yet been adequately explored (Greely, 2019).

While many countries and academic associations remain opposed to HGE, some academic associations have expressed, even before the He Jiankui affair, that it could be permissible under exceptional circumstances (Baylis et al., 2020). For example, the National Academies of Sciences, Engineering, and Medicine in the US took the stance that HGE clinical trials could be allowed in the future only if stringent criteria are met and for the prevention of severe diseases or conditions that lack viable alternatives (NASEM, 2017). Another example is a series of recommendations by the Nuffield Council on Bioethics, an independent body in the United Kingdom, which stated that HGE should be allowed only for purposes that are consistent with the welfare of a child who may be born, provided both safety and feasibility have been established (NCB, 2018). These cases indicate that HGE may be supported in the future to prevent severe diseases, provided the safety issues are overcome, and due consideration has been given to the welfare of future children.

Many have called for a public discussion on HGE involving various stakeholders (Scheufele et al., 2021). International organizations, including the International Society for Stem Cell Research (ISSCR), have highlighted the importance of discussions that involve a broad range of stakeholders (ISSCR, 2015). The Science Council of Japan, in its latest report, stressed the need for discussions that involve experts as well as diverse stakeholders among the lay public and designing a consensus-building process, given the issues concerning human dignity, eugenics, social discrimination, and impacts on future generations (SCJ, 2020).

Over ten surveys concerning HGE have been conducted overseas to address this need for discussion. Delhove and colleagues reviewed nine prior studies published between 2016 and March 2019 (Delhove et al., 2020). However, in many of these earlier surveys, respondents were often asked about their attitudes toward the use of the technology using questions such as: “Do you think somatic genome editing is acceptable for treating intractable genetic conditions?” or “Do you think genome editing in embryos is acceptable for avoiding genetic conditions that would cause substantial limitations in activities of daily living?” (STAT and Harvard T.H. Chan School of Public Health, 2016; McCauhey et al., 2016; Musunuru et al., 2017; Scheufele et al., 2017; Wang et al., 2017; Whitman et al., 2018). These questions were framed in this manner to address two issues: whether or not the use of genome editing in somatic cells or the germline is acceptable, and for what purposes it is acceptable. This would make it challenging for researchers to determine if a given answer reflects the respondent’s attitude toward genome editing in either somatic or germline cells or toward genome editing performed for specific purposes. Furthermore, the phrase “not acceptable” as a response would not sufficiently explain the respondent’s reasoning.

To date, two surveys have been conducted on HGE in Japan. First, a survey conducted by Uchiyama and colleagues on 10,881 laypeople and 937 patients suggested that the presence or absence of prior knowledge of genome editing and the level of such knowledge influenced attitudes toward HGE; specifically, genome-editing in embryos to treat life-threatening diseases or diseases that require long-term treatment (Uchiyama et al., 2018). The study, which included both laypeople and patients, provided noteworthy results in that the overall level of acceptance was higher in patients than in the general public and that their attitudes toward HGE varied depending on whether they had prior knowledge of it. In a second survey by Taguchi et al. (2019) genetic professionals, including 176 clinical genetics specialists, consulting genetics specialists, and 101 certified genetic counselors, were asked about their attitudes toward HGE and SGE. HGE formed the center of the discussion, and SGE is becoming increasingly accepted globally. The results suggested that HGE was more acceptable to treat a severe genetic disorder, although to a lesser degree than SGE. Nonetheless, these surveys have also been challenged by the framing of their questions.

To hold a societal discussion in pursuit of forming a consensus concerning HGE, we must understand the specific conditions under which the survey participants would consider HGE acceptable and the reasons for which they deem it unacceptable. This is because individual views on genome editing may be categorized into two fundamentally different groups: those opposed to all clinical applications of human genome editing and those opposed to editing certain targets and/or for certain purposes. Collecting responses that reflect such a broad range of views will help shape public discussions. Accordingly, we decided to assess the degree of acceptance for human genome editing in clinical applications on different targets and for different purposes. As it would be necessary to review and compare the views of a range of stakeholders to form a social consensus, we included members of the Japanese Society for Genome Editing in the survey to compare their attitudes with those of the laypeople, who were non-experts. Through this study, we aimed to elucidate the views held by laypeople (non-experts) and researchers (experts) on human genome editing in clinical applications, differences in their respective views, and the reasons for developing an attitude against its applications.

## 2 Materials and methods

### 2.1 Survey participants

Laypeople and researchers were surveyed in May 2019 (after the He Jiankui report). We worked with a private research company (GMO Research, Inc.) to develop an online survey platform and collect data after finalizing the survey design and the questionnaire. Registered members of the research company’s panel (aged 20–79) and members of the Japanese Society for Genome Editing were recruited to represent the laypeople and researchers, respectively. The sample size for the lay group was determined using a method employed in prior studies conducted on the Japanese general public (Akatsuka et al., 2021; Sawai et al., 2021) that assessed the degree of acceptance of *in vitro* gametogenesis technology. These studies utilized a three-point Likert scale and included approximately 3,000 participants. In our current study, we aimed to evaluate the degree of acceptance of genome editing under different circumstances using a similar three-point scale, namely, “unacceptable for any purpose,” “acceptable depending on the purpose,” and “acceptable for any purpose” (further details are provided in subsequent sections). Considering the increased number of questions and combinations of variables that needed to be analyzed in our survey compared to the aforementioned studies, we determined that a sample size of 4,000 individuals for the lay group may be more suitable. For this survey, the 4,000 laypeople were randomly divided into two groups: one was provided with information on genome editing, while the other was not. Basic information on genome editing was presented to the participants in the former group, followed by explanations and illustrations of the purposes of human genome editing and differences between SGE and HGE (please see Supplementary Material for details).

The company selected lay participants by asking for volunteers on its website using an opt-in sampling method (Sue and Ritter, 2007a). Participants were sampled to ensure that their sex and age distributions matched those of the Japanese demographics at the time of the survey (MIC, 2015). Specifically, in 2015 the Japanese population, used to estimate the sample size, was approximately 125 million, with approximately 61 million (48.9%) men and 64 million (51.2%) women. The age distribution consisted of 21 million (16.8%) individuals aged 19 and younger, 12 million (9.6%) individuals in their 20s, 15 million (12.0%) individuals in their 30s, 18 million (14.4%) individuals in their 40s, 15 million (12.0%) individuals in their 50s, 18 million (14.4%) individuals in their 60s, 13 million (10.4%) individuals in their 70s, and 9 million (7.2%) individuals aged 80 and older. Participants were compensated with an incentive equivalent to 29 JPY upon completing the questionnaire.

To recruit experts, a request to complete the survey was sent by e-mail to 335 researchers who were members of the Japanese Society for Genome Editing as of May 2019, with prior permission from the Society (Sue and Ritter, 2007b). As in the lay group, researchers were also sampled using an opt-in method (volunteer opt-in panels) (Sue and Ritter, 2007a); completing the survey was deemed as providing consent to participate. The researchers did not receive any remuneration for their participation.

### 2.2 Contents of the survey and process of developing a questionnaire

The data used in this article are part of the “Survey on Human Genome Editing of the Japanese General Public and Researchers Project.” The following groups of items were used in this survey (Supplementary Material for items 1 and 2 below).

1. Questions concerning the extent of scientific understanding (“literacy score,” see Supplementary Material S1)
2. Explanations on genome editing in general; explanations of human genome editing; questions concerning the extent of understanding of the explanations (only to the lay group provided with information, see Supplementary Material S2, 3)
3. Questions concerning attitudes to human genome editing in clinical applications (hereafter referred to as attitude questions)
4. Questions concerning participant attributes

**FIGURE 1:**
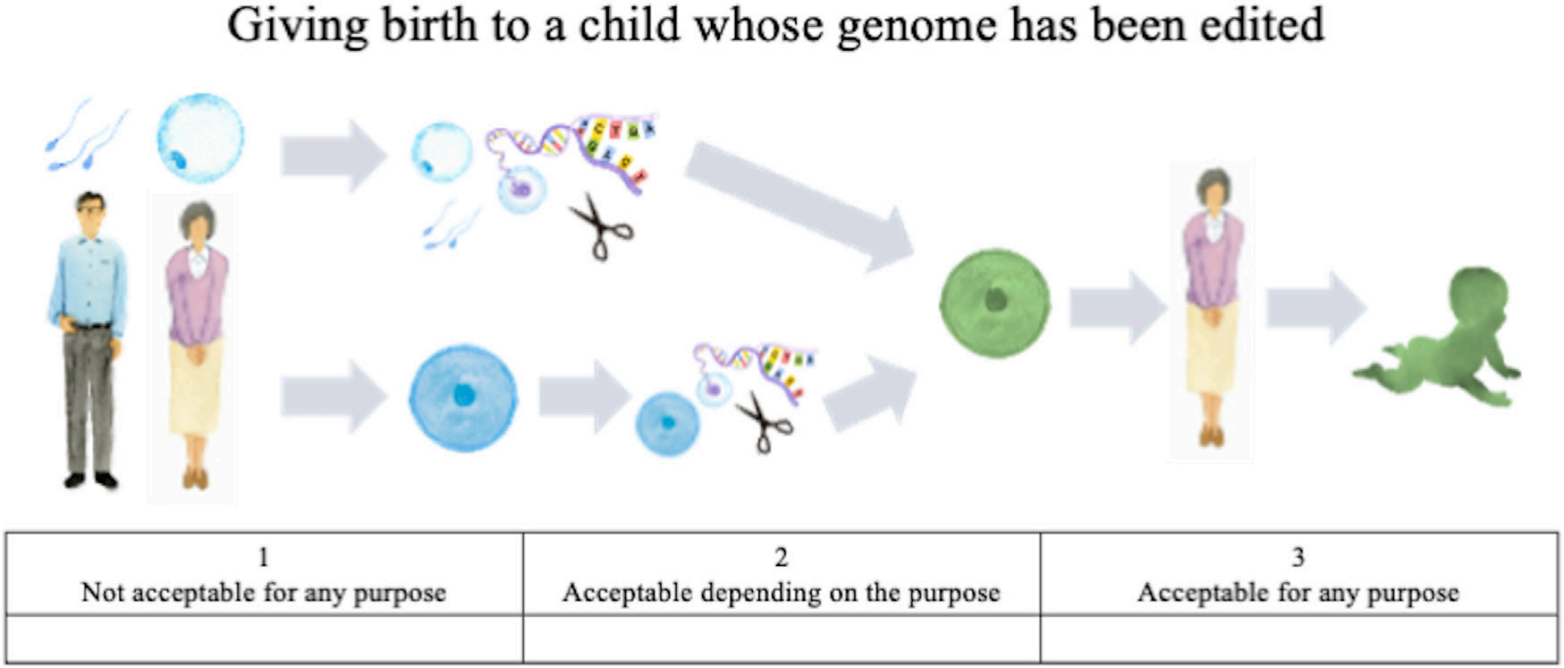
Illustration of genome editing of the germline in clinical applications provided to lay respondents.

**FIGURE 2:**
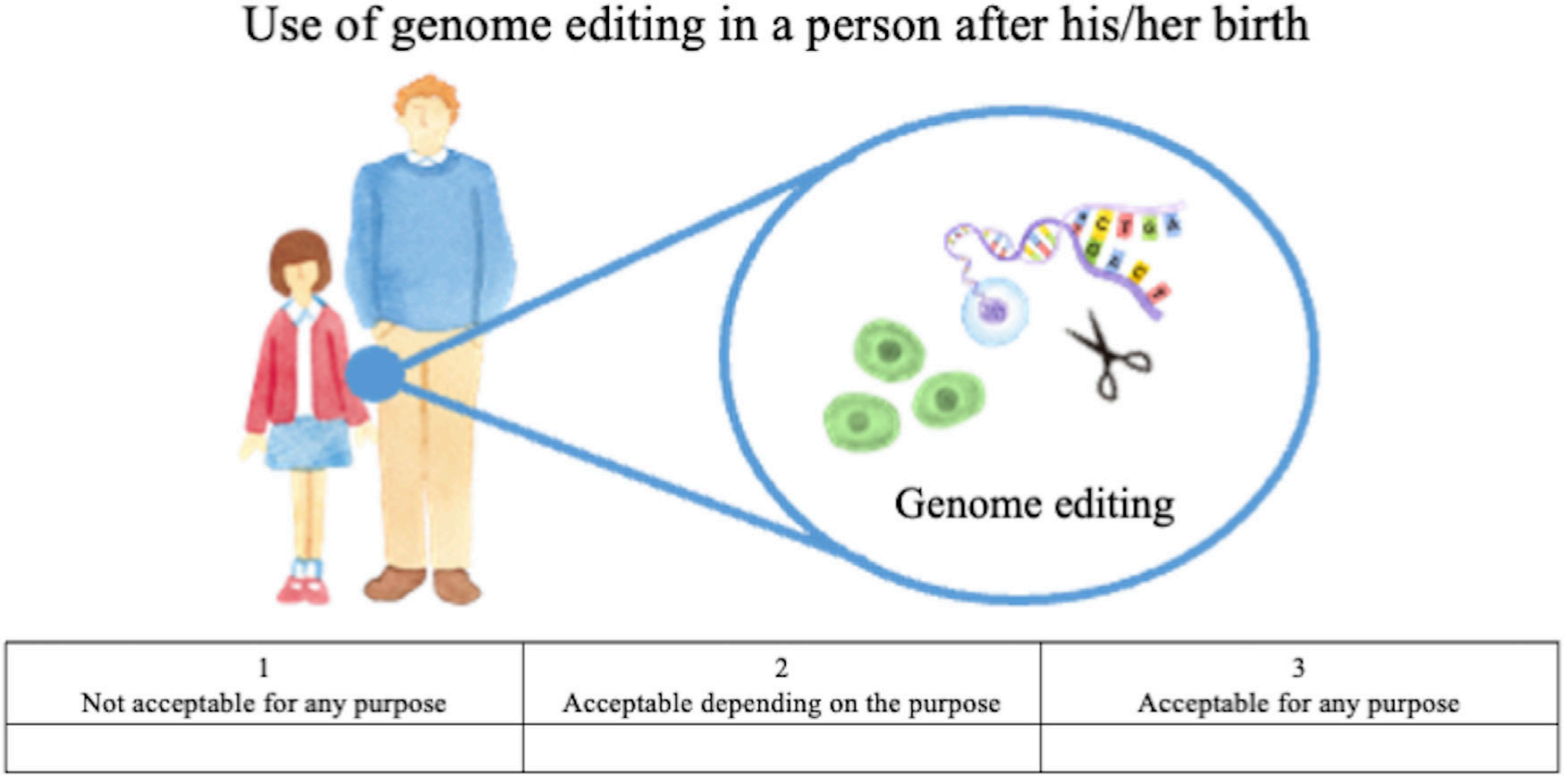
Illustration of genome editing of somatic cells in clinical applications provided to lay respondents.

Attitude questions included the following, each accompanied by illustrations:

• How do you personally feel about the prenatal use of genome editing not in research but in clinical medical applications, and a child whose genome has been edited being born? (Figure 1).
• How do you personally feel about using genome editing in a person after his/her birth, not in research but in clinical medical applications? (Figure 2).

The first question concerns HGE, that is, editing of germ cells or fertilized eggs and the genome-edited child being born, whereas the second concerns SGE, that is, editing of somatic cells of a person already born, child or adult. For both questions, the following choices were provided: “acceptable for any purpose,” “acceptable depending on the purpose,” and “not acceptable for any purpose.” These responses were scored on a Likert scale, with “acceptable for any purpose” scored as 3, “acceptable depending on the purpose” scored as 2, and “not acceptable for any purpose” scored as 1. For respondents who chose the second (acceptable depending on the purpose), an additional question was presented listing specific purposes; they were asked to select all options that might apply. These options were developed based on thirteen published surveys (STAT and Harvard 2016, Funk et al., 2016; Musunuru et al., 2017; Gaskell et al., 2017; Scheufele et al., 2017; Wang et al., 2017; Funk and Hefferon, 2018; Whitman et al., 2018; Uchiyama et al., 2018; Hendriks et al., 2018; Treleaven and Tuch, 2018; McCauhey et al., 2016; McCauhey et al., 2019), the bioethics literature on human genome editing (e.g., NASEM, 2015; NCB, 2016; NCB, 2018, WHO 2019), and policy discussions in Japan (COB, 2018; COB, 2019).

The list of specific purposes presented for HGE included nine options: “to allow (an infertile couple) to have a child” (hereafter, infertility treatment); “to cure a disease that may substantially shorten the life expectancy of the future child” (treatment of life-threatening diseases); “to cure a disease that may cause substantial limitations in daily and social activities of the future child” (treatment of debilitating diseases); “to prevent a disease that future child may develop (e.g., cancer, diabetes, HIV/AIDS)” (prevention of chronic diseases); “to make future child’s bones and muscles strong” (physical enhancement); “to have future child acquire high intelligence” (cognitive enhancement); “to make the future child less susceptible to obesity” (health enhancement); “to give future child personality traits ideal for parents” (personality enhancement), and “for other purposes” (other purposes).

For SGE, eight specific purposes were listed: “to cure a disease that causes substantial limitations in daily and social activities” (treatment of debilitating diseases); “to cure a disease that substantially shortens the life expectancy” (treatment of life-threatening diseases); “to prevent a disease that one may develop (e.g., cancer, diabetes, HIV/AIDS)” (prevention of chronic diseases); “to make one’s bones and muscles strong” (physical enhancement); “to acquire high intelligence” (cognitive enhancement); “to make one less susceptible to weight gain “(health enhancement); to “to make one’s personality traits ideal” (personality enhancement), and “for other purposes” (other purposes).

Questions concerning the level of scientific understanding were adapted from those used by Scheufele et al. to assess science literacy in genetics after obtaining due permission from Dr. Scheufele (Scheufele et al., 2018). To collect demographic data, laypeople and researchers were asked to provide their educational backgrounds, household income, religion, marital history, whether they wished to take a genetic test, and whether they had ever been treated for infertility. The researchers were asked additional questions concerning possession of a medical license and routine use of human specimens.

### 2.3 Data analysis

Non-parametric tests (Mann–Whitney U, Wilcoxon signed-rank, and chi-square) were performed to evaluate the results. To further assess the attitudes of laypeople who disapproved of genome editing, they were divided into two groups according to their answers to the attitude questions: “not acceptable for any purpose” versus those who chose either “acceptable for any purpose” or “acceptable depending on the purpose.” Binomial logistic regression was performed to assess the relationships between participant attributes and their attitudes toward genome editing. Among the demographic characteristics, we excluded respondents who chose “Undisclosed” or “I do not know” for experience with infertility treatment, household income, religious affiliation, genetic testing, and serious illness from the analysis. We replaced respondent age with age range categories (20–29, … , 70–79) and included it as an explanatory variable in the regression equation. For multivariable logistic regression, explanatory variables were entered using the forced-entry method, and the variance inflation factor (VIF) was confirmed to be < 10 for each explanatory variable to avoid multicollinearity (O’Brien, 2007).

Statistical significance was set at *p* < 0.01. In nonparametric tests, the effect size was calculated according to Cohen (1988); *r* = 0.10 represented small; *r* = 0.30 medium; and r = 0.50 large effects. Analyses were performed using IBM SPSS Regression 27.0 (IBM Corp., NY, United States) and Microsoft Excel for Mac 16.54 (Microsoft Corp., WA, United States).

## 3 Results

### 3.1 Participant characteristics

Demographic characteristics are presented in Table 1. Responses were collected from 4,424 members of the research company panel representing the laypeople. Among these, 2,235 were provided with information, whereas 2,189 controls were not provided any information; their sex and age distributions approximately matched those of the Japanese public. The response rate of the lay group, who were recruited from the research company’s panel, was unknown. For the researchers, responses were collected from 98 of the 335 (29.3%) members of the Japanese Society for Genome Editing. For the attitude question items, no difference was observed between laypeople provided with information and those without (*p* = 0.45 in Attitudes toward HGE; *p* = 0.02 in Attitudes toward SGE); therefore, all the lay participants were combined and treated as a single group. Responses to the questions concerning the level of scientific understanding (“literacy score”) are summarized in Supplementary Table S1.

**TABLE 1:** Respondents’ demographic characteristics.

	Laypeople (*n* = 4424)		Researchers (*n* = 98)	
	n	%	n	%
Sex				
Male	2,198	49.7	80	81.6
Female	2,226	50.3	18	18.4
Age				
20–29	563	12.7	11	11.2
30–39	727	16.4	27	27.6
40–49	861	19.5	29	29.6
50–59	730	16.5	25	25.5
60–69	877	19.8	5	5.1
70–79	666	15.1	1	1.0
Marital status				
Married	2,951	66.7	70	71.4
Unmarried	1,473	33.3	28	28.6
Presence or absence of children				
Yes	2,390	54.0	58	59.2
No	2,034	46.0	40	40.8
Experience of infertility treatment				
Yes	273	6.2	15	15.3
No	3,784	85.5	76	77.6
I do not know	283	6.4	4	4.1
Undisclosed	84	1.9	3	3.1
Educational Background				
Elementary school	2	0.0	0	0.0
Junior high school	131	3.0	0	0.0
High school	1,335	30.2	1	1.0
Technical college	452	10.2	0	0.0
Two-year college	448	10.1	0	0.0
Four-year college	1,878	42.5	12	12.2
Postgraduate studies (master’s degree)	136	3.1	20	20.4
Postgraduate studies (doctorate)	42	0.9	65	66.3
Household income [yen/year]				
Less than two million	451	10.2	1	1.0
Two to four million	1037	23.4	10	10.2
Four to six million	929	21.0	12	12.2
Six to eight million	572	12.9	20	20.4
Eight to ten million	345	7.8	16	16.3
Over ten million	404	9.1	25	25.5
Undisclosed	686	15.5	14	14.3
Religious Affiliation				
Yes	567	12.8	73	74.5
No	3607	81.5	14	14.3
Undisclosed	250	5.7	11	11.2
What is your present religion?				
Christian	75	1.7	0	0.0
Buddhist	392	8.9	12	12.2
Islam	1	0.0	0	0.0
Shinto	43	1.0	1	1.0
Hindu	0	0.0	0	0.0
Others	21	0.5	1	1.0
Undisclosed	35	0.8	0	0.0
Would you like to take a genetic test that can predict the likelihood of diseases you may get in the future (e.g., cancer, diabetes)?				
Yes	1396	31.6	56	57.1
No	1457	32.9	25	25.5
I do not know	1571	35.5	17	17.3
Have you or a family member had a serious illness (e.g., cancer, diabetes, heart disease, stroke, pneumonia)?				
Yes	1,911	43.2	56	57.1
No	1,968	44.5	33	33.7
I do not know	394	8.9	5	5.1
Undisclosed	151	3.4	4	4.1
Do you have a medical license?				
Yes	―	―	9	9.2
No	―	―	89	90.8
Do you usually conduct research using human samples?				
Yes	―	―	33	33.7
No	―	―	65	66.3

### 3.2 Attitudes toward HGE

**FIGURE 3:**
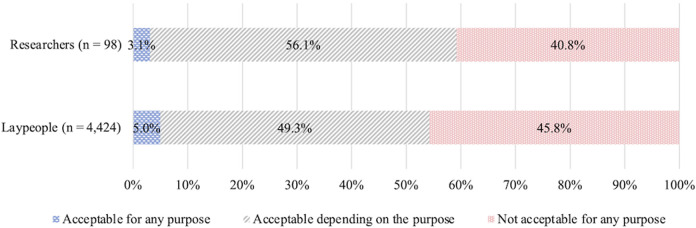
Attitudes toward clinical, prenatal HGE.

No differences in the distributions of responses were observed between laypeople and researchers (*Z* = −0.70, *p* = 0.48, *r* = 0.01). The percentage of respondents choosing each option was, from largest to smallest, “acceptable depending on purpose” (laypeople 49.3%; researchers 56.1%), “not acceptable for any purpose” (laypeople 45.8%; researchers 40.8%), and “acceptable for any purpose” (laypeople 5.0%; researchers 3.1%) (Figure 3). These percentages may not add up to 100% due to rounding from the second decimal place.

To describe the characteristics of the lay group who chose “not acceptable for any purpose,” multivariate binomial logistic regression was performed (Cox and Snell *R* ^2^ = 0.09, VIF < 2.50, Table 2). The following attributes were statistically significant: female, older individuals, and reluctance to take genetic testing (odds ratio were 0.73, 1.22, and 0.45, respectively; Table 2).

**TABLE 2:** Demographics characteristics of the respondents who do not accept genome editing of the germline in clinical applications at all (*N* = 4,424).

Demographic characteristics	Univariate binomial logistic regression analysis									Multivariate binomial logistic regression analysis^,^ ^b^ ^,^ ^c^							
	*B*	*SE B*	*Wald*	*OR*	*95% CI*		*df*	*P*	*n* ^a^	*B*	*SE B*	*Wald*	*OR*	*95% CI*		*df*	*P*
					*LL*	*UL*								*LL*	*UL*		
Male	−0.31	0.06	25.71	0.74	0.65	0.83	1	0.00	4424	−0.31	0.09	11.36	0.73	0.61	0.88	1	0.00
Age (10-year range)	0.26	0.02	184.61	1.30	1.25	1.35	1	0.00	4424	0.20	0.03	33.16	1.22	1.14	1.30	1	0.00
Literacy Score	0.05	0.01	18.75	1.05	1.03	1.08	1	0.00	4424	0.04	0.02	4.48	1.04	1.00	1.08	1	0.03
Educational background	0.00	0.02	0.04	1.00	0.96	1.04	1	0.83	4424	0.02	0.03	0.51	1.02	0.96	1.09	1	0.48
Household income	−0.03	0.02	1.46	0.97	0.93	1.02	1	0.23	3738	0.01	0.03	0.05	1.01	0.95	1.07	1	0.83
Have religious affiliation	0.03	0.09	0.13	1.03	0.87	1.23	1	0.72	4174	−0.20	0.13	2.50	0.82	0.64	1.05	1	0.11
Married	0.57	0.07	76.49	1.77	1.56	2.02	1	0.00	4424	0.07	0.15	0.18	1.07	0.79	1.44	1	0.67
Have child(ren)	0.47	0.06	59.80	1.60	1.42	1.81	1	0.00	4424	0.20	0.13	2.28	1.23	0.94	1.59	1	0.13
Interested in taking genetic testing	−0.87	0.08	123.45	0.42	0.36	0.49	1	0.00	2853	−0.81	0.09	74.42	0.45	0.37	0.54	1	0.00
Have a serious illness	0.23	0.06	12.92	1.26	1.11	1.43	1	0.00	3879	0.20	0.09	4.95	1.23	1.03	1.47	1	0.03
Have undergone infertility treatment	−0.28	0.13	4.89	0.75	0.59	0.97	1	0.03	4057	−0.34	0.17	3.98	0.71	0.51	0.99	1	0.05

^a^ The number of respondents who were included in the univariate analysis.

^b^ There were 2,305 respondents included in the multivariate analysis.

^c^ The coefficients of determination, Cox-Snell *R* ^2^ = 0.09, Nagelkerke *R* ^2^ = 0.12.

B, partial regression coefficient; OR, odds ratio; CI, confidence interval; LL, lower limit; UL, upper limit; *R* ^2^, the coefficients of determination.

### 3.3 Attitudes toward specific purposes among respondents who chose “acceptable depending on purpose” for HGE

**FIGURE 4:**
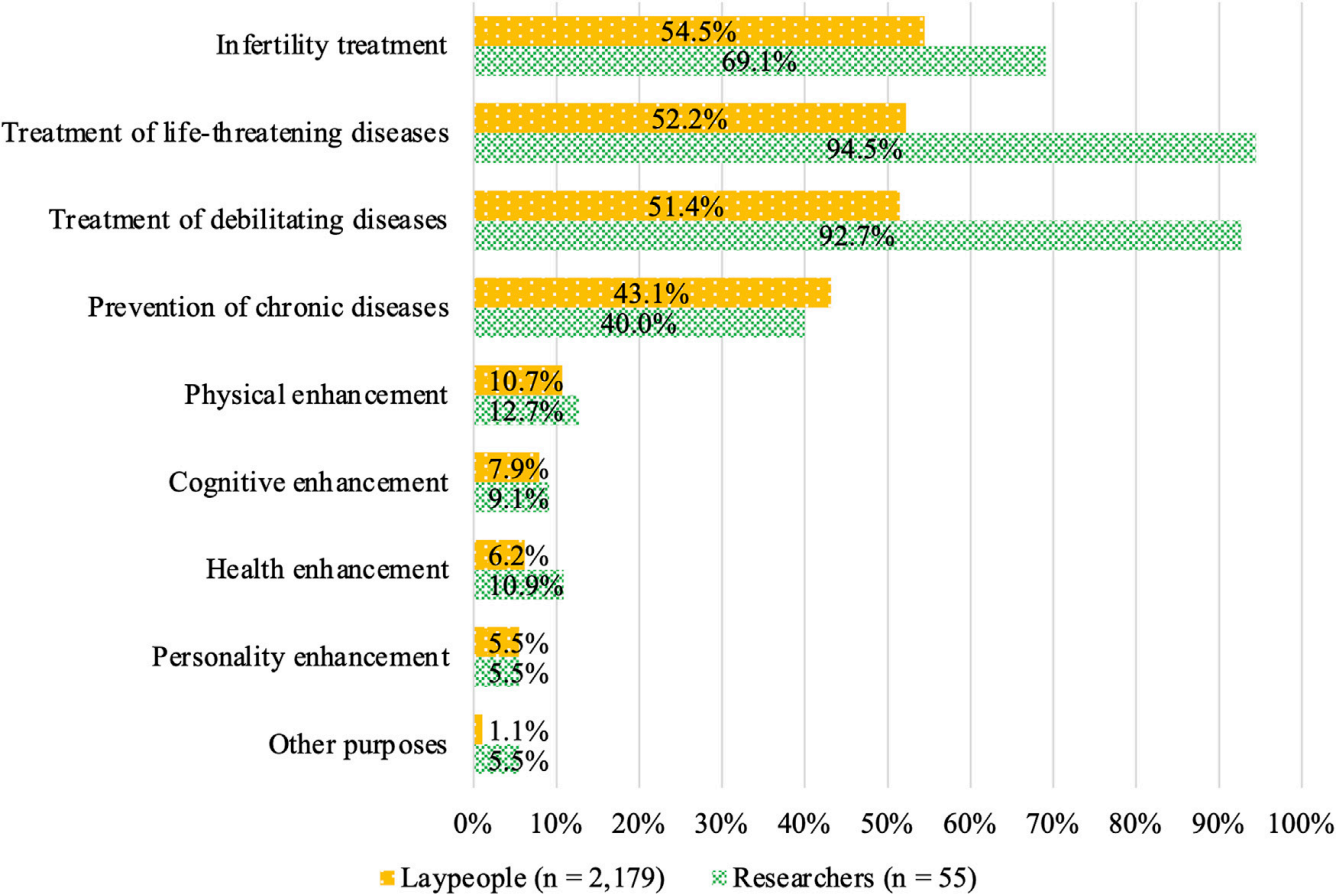
Attitudes toward specific HGE purposes among respondents who chose “acceptable depending on purpose.”

Acceptance among laypeople was shown in Figure 4, with most of the respondents finding the following acceptable: infertility treatment (54.5%), treatment of life-threatening diseases (52.2%), and treatment of debilitating diseases (51.4%) (Figure 4). Meanwhile, their acceptance of enhancement purposes was 10.7, 7.9, 6.2, and 5.5% for physical, cognitive, health, and personality enhancements, respectively. The acceptance rate was 1.1% for other purposes. Acceptance by researchers was 94.5% and 92.7% for the treatment of life-threatening and debilitating diseases, respectively, compared with 69.1% for infertility treatment. The acceptance of enhancement purposes among researchers was similar to that of lay participants, with 12.7, 9.1, 10.9, and 5.5% for physical, cognitive, health, and personality enhancement, respectively. The acceptance rate was 5.5% for other purposes.

A comparison of responses to the attitude-related questions between laypeople and researchers suggested that the degree of acceptance was substantially higher among the researchers for treating life-threatening and debilitating diseases as well as infertility. Meanwhile, acceptance for the prevention of chronic diseases did not significantly differ from that for treating diseases, namely, life-threatening and debilitating diseases, among laypeople. In contrast, the researchers were less accepting of editing for the prevention of chronic diseases than for treating diseases.

### 3.4 Attitudes toward SGE

**FIGURE 5:**
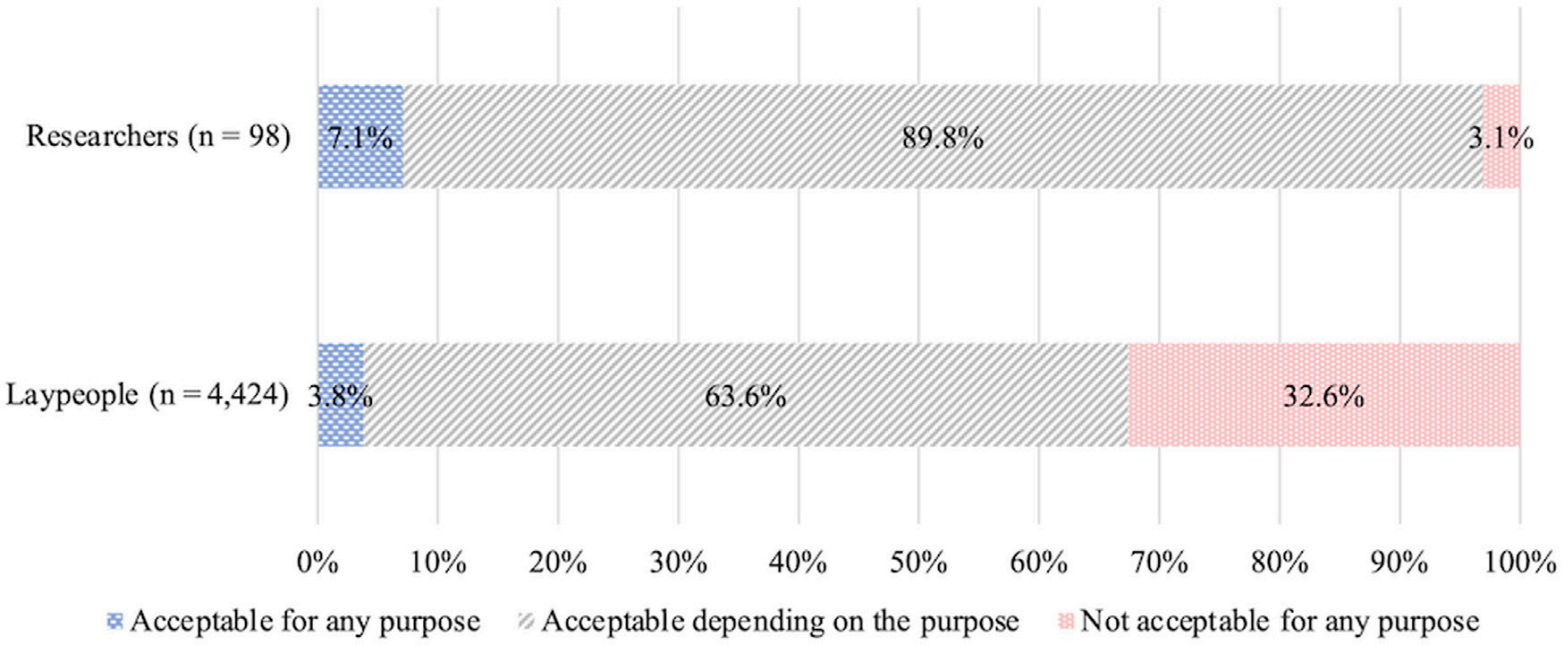
Acceptance of clinical SGE.

Among laypeople, the responses were as follows: “acceptable depending on purpose” (63.6%), “not acceptable for any purpose” (32.6%), and “acceptable for any purpose” (3.8%). Unlike the trends observed with HGE, acceptance was significantly higher for SGE (*Z* = 15.77, *p* ≤ 0.001, *r* = 0.24) (Figure 5).

The percentage of researchers choosing each option was: “acceptable depending on purpose” (89.8%), “acceptable for any purpose” (7.1%), and “not acceptable for any purpose” (3.1%). Compared to their attitudes toward HGE, the percentage of respondents choosing “not acceptable for any purpose” was lower; many chose “acceptable depending on the purpose.”

Multivariable binomial logistic regression was performed to assess the characteristics of laypeople who chose “not acceptable for any purpose” (Cox and Snell *R* ^2^ = 0.05, VIF < 2.50, Table 3). The following attributes exhibited statistical significance: older individuals, low literacy and reluctance to take genetic testing (odds ratio were 0.93 and 0.46, respectively; Table 3).

**TABLE 3:** Demographics characteristics of the respondents who do not accept genome editing of somatic cells in clinical applications at all (*N* = 4,424).

Demographic characteristics	Univariate binomial logistic regression analysis									Multivariate binomial logistic regression analysis^,^ ^b^ ^,^ ^c^							
	*B*	*SE B*	*Wald*	*OR*	*95% CI*		*df*	*P*	*n* ^a^	*B*	*SE B*	*Wald*	*OR*	*95% CI*		*df*	*P*
					*LL*	*UL*								*LL*	*UL*		
Male	−0.28	0.06	18.68	0.76	0.67	0.86	1	0.00	4424	−0.19	0.10	3.65	0.83	0.69	1.01	1	0.06
Age (10-year range)	0.14	0.02	49.14	1.15	1.11	1.20	1	0.00	4424	0.09	0.04	6.58	1.10	1.02	1.18	1	0.01
Literacy Score	−0.07	0.01	26.76	0.94	0.91	0.96	1	0.00	4424	−0.07	0.02	12.09	0.93	0.90	0.97	1	0.00
Educational background	−0.05	0.02	4.77	0.95	0.91	1.00	1	0.03	4424	0.01	0.04	0.06	1.01	0.94	1.08	1	0.82
Household income	−0.01	0.02	0.20	0.99	0.95	1.04	1	0.65	3738	0.01	0.03	0.03	1.01	0.94	1.08	1	0.86
Have religious affiliation	0.03	0.10	0.08	1.03	0.85	1.24	1	0.78	4174	−0.04	0.13	0.09	0.96	0.74	1.25	1	0.76
Married	0.42	0.07	34.94	1.52	1.32	1.74	1	0.00	4424	0.12	0.16	0.57	1.13	0.82	1.56	1	0.45
Have child(ren)	0.31	0.07	23.22	1.37	1.20	1.55	1	0.00	4424	0.10	0.14	0.45	1.10	0.83	1.45	1	0.51
Interested in taking genetic testing	−0.86	0.08	108.45	0.43	0.36	0.50	1	0.00	2853	−0.78	0.10	64.14	0.46	0.38	0.56	1	0.00
Have a serious illness	−0.06	0.07	0.82	0.94	0.82	1.08	1	0.37	3879	−0.04	0.10	0.21	0.96	0.79	1.16	1	0.65
Have undergone infertility treatment	0.02	0.13	0.03	1.02	0.79	1.33	1	0.86	4057	−0.11	0.18	0.38	0.89	0.63	1.28	1	0.54

^a^ The number of respondents who were included in the univariate analysis.

^b^ There were 2,305 respondents included in the multivariate analysis.

^c^ The coefficients of determination, Cox-Snell *R* ^2^ = 0.05, Nagelkerke *R* ^2^ = 0.08.

B, partial regression coefficient; OR, odds ratio; CI, confidence interval; LL, lower limit; UL, upper limit; *R* ^2^, the coefficients of determination.

### 3.5 Attitudes toward specific SGE purposes among respondents who chose “acceptable depending on purpose”

**FIGURE 6:**
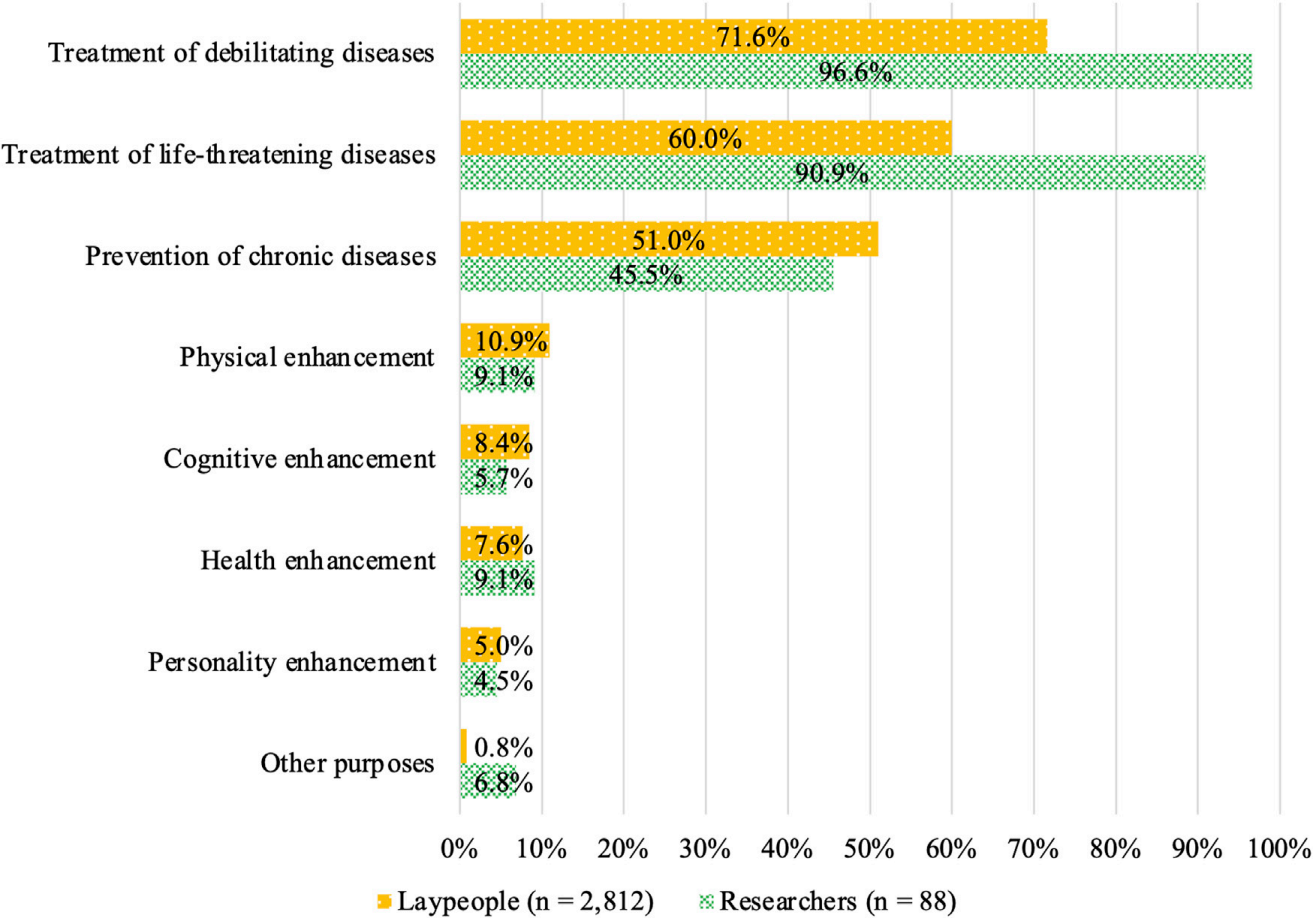
Attitudes toward specific SGE purposes among respondents who chose “acceptable depending on purpose.”

Acceptance among laypeople was shown in Figure 6. It was higher for the treatment of debilitating diseases (71.6%), treatment of life-threatening diseases (60.0%), and prevention of chronic diseases (51.0%) compared with the other options (Figure 6). In contrast, acceptance of SGE for enhancement was 10.9, 8.4, 7.6, and 5.0% for physical, cognitive, health, and personality enhancement, respectively. The acceptance rate was 0.8% for other purposes. The acceptance among expert participants varied, even among the disease-related purposes; it was higher for the reatment of debilitating diseases (96.6%) and the treatment of life-threatening diseases (90.9%) than it was for the prevention of chronic diseases (45.5%). The acceptance of enhancement purposes among researchers was similar to that among the lay participants, with 9.1, 5.7, 9.1, and 4.5% for physical, cognitive, health, and personality enhancement, respectively. The acceptance rate was 6.8% for other purposes.

## 4 Discussion

### 4.1 Lay and expert attitudes depend on editing targets and purposes

Comparing the distribution of respondents who chose “not acceptable for any purpose” for HGE and SGE indicated a slight difference in the acceptance of both among laypeople and a substantial difference among the researchers. It also suggests that laypeople and researchers are more concerned about HGE than SGE.

While differences in question forms preclude an exact comparison, our data have much in common with those from several prior surveys concerning HGE and SGE. For example, a survey of 301 attendees at an American Heart Association conference suggested that acceptance of using genome editing to avoid the risk of serious diseases was lower for HGE than for SGE (Musunuru et al., 2017). Another survey of approximately 1,000 laypeople from 11 countries in Europe and North America reported that acceptance was lower for HGE than for SGE for treating disease (Gaskell et al., 2017). Similarly, our study indicated that acceptance was lower for HGE than for SGE.

Some prior surveys have indicated that attitudes depend more on the purposes of interventions than on their targets. For example, a survey of 10,067 social-media users and another conducted on 1,600 US laypeople both reported that attitudes toward genome editing were influenced more by purpose, such as treatment versus enhancement, than by differences in target, such as somatic cells versus the germline (McCauhey et al., 2016; Scheufele et al., 2017). In our study, “acceptable depending on purpose” was the most common choice for both HGE and SGE, with acceptance higher for treatment than for enhancement. These data suggest, as prior surveys did, that the intervention target alone does not determine attitudes to human genome editing.

Many past surveys asked participants about targets and purposes in a single question, making it challenging to determine which was more important. However, we surveyed attitudes regarding targets and purposes separately, which better indicated how they influenced attitudes. This implies that dichotomous positions, such as “SGE is always acceptable, while HGE is always unacceptable” or “human genome editing is always acceptable for treatment, but always unacceptable for other purposes,” would not be supported, at least not in Japan.

Notably, the choice regarding attitudes toward HGE in this study, “not acceptable for any purpose,” was more common in women than in men, in older than in younger respondents, and in those who were more reluctant than willing to take genetic testing. While several prior studies have shown that women are more inclined to oppose gene therapy, it has also become apparent that age influences acceptance (Delhove et al., 2020). Considering our finding that those who were reluctant to take a genetic test were more averse to HGE, one may argue that people who are averse to an act of interference with genes by humans, or those who are not interested in such an act in the first place, are likely to be inclined to oppose HGE.

### 4.2 Laypeople and researchers often distinguish between treatment and enhancement

As noted in the earlier section, we observed that acceptance of genome editing varied depending on whether the target is a germline or a somatic cell. We also demonstrated that acceptance depended on whether the purpose was for treatment or enhancement. Specifically, laypeople who chose “acceptable depending on purpose” in the attitude questions on SGE and HGE were likely to see genome editing for treating disease as more acceptable than that for other purposes, including enhancement.

Several factors may explain these findings. With disease treatment as the purpose of the intervention, for example, it might have been easy for laypeople to imagine a real person suffering from a real disease, or they may have found no reason to object to disease treatment regardless of means, that is, whether or not it is genome editing. Conversely, intervention for enhancement purposes likely did not inspire the respondents to think of individuals suffering the same way as disease treatment did. With HGE, safety issues and other concerns may have led the respondents to decide that the risk-benefit tradeoffs were unacceptable. They may also have determined the acceptability of the enhancement relative to disease treatment.

The prevention of chronic diseases, including treatment and enhancement elements, was slightly less acceptable than disease treatment for SGE and HGE. Laypeople who found disease treatment acceptable but the prevention of chronic diseases unacceptable may presumably have judged that the necessities for the prevention of chronic diseases were not as crucial as those for disease treatment. In this study, genome editing to prevent HIV transmission (performed by He Jiankui) was presented as an example of genome editing to prevent chronic disease. Less than half of those who chose “acceptable depending on purpose” approved of this option. In our true/false questionnaire concerning the act committed by He Jiankui, only 31.9% of lay participants were aware of it (Supplementary Table S2). Despite the lack of familiarity with his act in Japan, many Japanese people would likely not endorse it judging by the attitudes toward the prevention of chronic diseases among the respondents of this study.

The responses of the researchers exhibited an overall trend similar to that among laypeople in that their acceptance was higher for genome editing for disease treatment than that for enhancement. However, relative to laypeople, the gap between the degree of acceptance for disease treatment and that for the prevention of chronic diseases was more pronounced. A possible reason for this difference is that the researchers, who have improved knowledge of genome editing, may have weighed the feasibility and scientific validity of such treatments. Specifically, they may have reasoned that genome editing could be a viable option for the treatment of life-threatening or debilitating diseases caused by specific gene mutations while questioning the scientific validity of using genome editing to address lifestyle-related diseases, such as cancer and diabetes, at a stage where it has not yet been developed. The availability of alternate modes of preventing HIV transmission was highlighted in the wake of the announcement by He Jiankui (e.g., NASEM, 2019), and the researchers likely concluded that there was no pressing need for genome editing. The Japanese Society for Genome Editing voiced its concerns immediately following his announcement, stating that his act was ethically unacceptable (JSGE, 2018). The percentage of correct answers for the aforementioned true/false questionnaire concerning this report was 97.9% among the researchers (Supplementary Table S3).

We observed that the acceptance for genome editing for enhancement was significantly lower than that for therapeutic purposes, consistent with all prior surveys published to date, including an opinion survey of Japanese clinical genetics specialists, consulting genetics specialists, and certified genetic counselors (McCauhey et al., 2016; Gaskell et al., 2017; Musunuru et al., 2017; Scheufele et al., 2017; Wang et al., 2017; Funk and Hefferon, 2018; Taguchi et al., 2019). This suggests that many laypeople and researchers in Japan recognize ethical differences between treatment and enhancement. Therefore, neither SGE nor HGE is likely to garner support in Japan when performed for enhancement.

### 4.3 Most laypeople have a negative attitude toward clinical genome editing

In this study, approximately 30%–45% of laypeople chose “not acceptable for any purpose” as their attitude toward SGE and HGE. This suggests that a certain portion of the public is averse to clinical genome editing, regardless of whether it targets somatic or germline cells. Of the respondents who chose “acceptable depending on the purpose,” 50%–75% said SGE or HGE was acceptable for treating diseases. Conversely, most lay people were not inclined to embrace SGE or HGE.

While the survey conducted by Uchiyama et al. found that around half of laypeople, and approximately 30% of patients, considered HGE unacceptable under any circumstances (Uchiyama et al., 2018), we observed that a considerably greater proportion of laypeople had negative attitudes toward both SGE and HGE. One possible reason for this may be that illustrations depicting the workflow of SGE and HGE provided to participants would have aided enhanced visualization. Another reason is that participants were asked about possible expectations and concerns surrounding SGE and HGE before they answered attitude questions, which may have made them aware of some controversy regarding these practices. The controversies may be associated with safety risks, such as undesirable effects on future generations. Some participants may also have had an issue with the act of manipulating genes, viewing it as “unnatural” or “playing God,” as has often been noted in conventional bioethical discussions (e.g., van Dijke et al., 2018).

In another Japanese survey, 3,000 laypeople and 197 experts were surveyed to understand their attitudes toward genome editing technologies, gene-edited food, and other products, using questions on perceptions of anxiety. Approximately half of laypeople agreed with the statement “bioethically questionable” (i.e., they chose “agree” or “agree somewhat”) or “cannot understand well and feel somewhat fearful” (i.e., they chose “agree” or “agree somewhat”) (Tachikawa et al., 2017). Conversely, among the experts, approximately 30% and 10% agreed with the statements “bioethically questionable” and “cannot understand well and feel somewhat fearful,” respectively (Tachikawa et al., 2017). Although comparing the results of the survey by Tachikawa et al. with those of our study would be inappropriate, we speculate that a certain proportion of laypeople felt anxiety over HGE as well as genome editing in general. This suggests a potentially large gap between laypeople and researchers in anxiety and technological concerns, which should be considered when determining how to shape public discussions.

### 4.4 Limitations and significance of the study

In past surveys concerning SGE and HGE, it was often unclear whether targets (i.e., somatic cells versus the germline) or purposes (i.e., therapeutic versus non-therapeutic) influenced attitudes toward human genome editing. Our focus was to determine whether the attitudes of laypeople and researchers in Japan would vary depending on targets and purposes; both influenced attitudes.

However, the structure of our questionnaire likely affected attitudes toward human genome editing. For example, we did not present specific examples of different intervention purposes to respondents who chose “acceptable for any purpose” or “not acceptable for any purpose” concerning HGE and SGE. The respondents may have expressed different attitudes if presented with specific examples. Nonetheless, one objective of our study was to identify respondents who found the use of genome editing technologies acceptable or unacceptable regardless of purpose; our choice not to present specific purposes may represent both a limitation and a strategic choice to differentiate this from prior studies.

Our expert respondents were limited to members of an academic association concerned with genome editing. The results may differ if physicians and scientists specializing in genetic disorders and/or reproductive medicine are surveyed. These warrant continued efforts to conduct research on human genome editing among a broad spectrum of stakeholders.

Finally, we used lay members of a research company’s panel. Given the possibility that such members have higher literacy than the lay public, this choice could provide significant selection bias. It is crucial to note that our survey results may differ slightly if different subjects (with an equivalent literacy level to the lay public) had been involved.

## 5 Conclusion

Our study yielded three key findings concerning human genome editing in clinical applications. First, HGE is far from being widely embraced by the laypeople, even in treating life-threatening or debilitating diseases. It is also considered controversial even among researchers, given that as much as 40% of them regarded HGE as being “not acceptable for any purpose,” even though over 90% of the researchers who chose “acceptable depending on purpose” in the HGE questions approved of HGE for therapeutic purposes. Second, expert support for SGE is lower than that of laypeople; however, researchers support it for treating life-threatening or debilitating diseases. The attitudes of the laypeople can eventually change depending on possible new technological innovations, the availability of alternative strategies, and risk assessments. Nonetheless, public discussions on bioethical issues concerning human genome editing, in general, must be conducted to address the anxiety over its clinical applications. Third, securing support for human genome editing for enhancement among laypeople and researchers in Japan will be challenging. The meaning of “enhancement” can vary depending on how it is defined relative to therapy. Nonetheless, one cannot rule out the possibility that an act currently considered to fall outside the definition of therapy may be viewed as therapy and accepted in the future. In the past, debates regarding the acceptability of human genome editing in clinical applications have often centered on HGE in many countries. Society must now debate the acceptability of human genome editing, including HGE and SGE.

## Data Availability

The raw data supporting the conclusion of this article will be made available by the authors, without undue reservation.
