# Supplementary material for: Human genome editing in clinical applications: Japanese lay and expert attitudes
Source: Front Genet. 2023 Aug 17;14:1205092. doi: 10.3389/fgene.2023.1205092 (PMC10469609; doi:10.3389/fgene.2023.1205092)
Supplement: Supplementary file 1 [file DataSheet2.zip › Supplementary_Materials/Supplemental Table 2.pdf]

Supplemental Table 2. Grasp of scientific understanding among the general public (n = 4424) and percentage of correct answers

	Definitely false	Probably false	I do not know	Probably true	Definitely true	% correct answers
1. Over time, human beings have incorporated some DNA of different species of animals and viruses into their own.	163	500	2286	1350	125	33.3
2. Personal behavior or environmental factors do not change human DNA sequences.	101	746	1859	1366	352	19.2
3. Tomatoes do not normally carry any gene, while genome-edited tomatoes do.	399	561	2812	613	39	21.7
4. Scientists have rewritten over 30 genetic features of commercially available plants so far using genome editing technologies.	26	152	2910	1224	112	30.2
5. Genome-edited crops can be legally grown in all of Europe.	77	521	3101	688	37	13.5
6. Genome-edited produce is currently sold at supermarkets.	115	634	2464	1105	106	27.4
7. Scientists have never used genome editing in humans to date.	329	1195	2231	564	105	34.4
8. In Japan, the government (Cabinet Office) is engaged in an ongoing debate about genome editing.	49	257	2612	1363	143	34
9. Human beings have evolved from primitive organisms.	60	171	1585	1916	692	58.9
10. There were reports in 2018 on the birth of babies through genome editing in fertilized eggs.	59	309	2645	1039	372	31.9

The percentage of correct answers for each item was based on the total number of respondents who answered each item correctly. E.g., for point number 10. There were reports in 2018 on the birth of babies through genome editing in fertilized eggs," the answers "Probably true" and "Definitely true" counted as being correct.
