# Supplementary material for: Human genome editing in clinical applications: Japanese lay and expert attitudes
Source: Front Genet. 2023 Aug 17;14:1205092. doi: 10.3389/fgene.2023.1205092 (PMC10469609; doi:10.3389/fgene.2023.1205092)
Supplement: Supplementary file 1 [file DataSheet2.zip › Supplementary_Materials/Supplemental Table 3.pdf]

Supplemental Table 3. Grasp of scientific understanding among researchers (n = 98) and percentage of correct answers

	Definitely false	Probably false	I do not know	Probably true	Definitely true	% correct answers
1. Over time, human beings have incorporated some DNA of different species of animals and viruses into their own.	0	1	6	52	39	92.9
2. Personal behavior or environmental factors do not change human DNA sequences.	33	41	8	12	4	75.5
3. Tomatoes do not normally carry any gene, while genome-edited tomatoes do.	84	7	4	2	1	92.8
4. Scientists have rewritten over 30 genetic features of commercially available plants so far using genome editing technologies.	5	14	37	36	6	42.8
5. Genome-edited crops can be legally grown in all of Europe.	15	43	30	10	0	59.2
6. Genome-edited produce is currently sold at supermarkets.	18	40	19	20	1	21.4
7. Scientists have never used genome editing in humans to date.	54	35	4	4	1	90.8
8. In Japan, the government (Cabinet Office) is engaged in an ongoing debate about genome editing.	0	2	6	49	41	91.8
9. Human beings have evolved from primitive organisms.	0	1	8	54	35	90.8
10. There were reports in 2018 on the birth of babies through genome editing in fertilized eggs.	1	0	1	26	70	97.9

The percentage of correct answers for each item was based on the total number of respondents who answered each item correctly. E.g., for point number 10. There were reports in 2018 on the birth of babies through genome editing in fertilized eggs," the answers "Probably true" and "Definitely true" counted as being correct.
