# Supplementary material for: Human genome editing in clinical applications: Japanese lay and expert attitudes
Source: Front Genet. 2023 Aug 17;14:1205092. doi: 10.3389/fgene.2023.1205092 (PMC10469609; doi:10.3389/fgene.2023.1205092)
Supplement: Supplementary file 2 [file DataSheet1.DOCX]

Supplementary Material

Human Genome Editing in Clinical Applications: Japanese Lay and Expert Attitudes

Tsutomu Sawai, Taichi Hatta, Kyoko Akatsuka, Misao Fujita

*** Correspondence:** Misao Fujita: misao-fujita@cira.kyoto-u.ac.jp

# Supplementary Information

Supplemental Information 1. Understanding of the science

Supplemental Information 2. Explanations on Genome editing

Supplemental Information 3. Explanations on Genome editing in humans

# Supplementary Table

Supplemental Table 1. Scientific understanding of the respondents (Literacy Score)

Supplemental Table 2. Grasp of scientific understanding among the general public (n = 4424) and percentage of correct answers

Supplemental Table 3. Grasp of scientific understanding among researchers (n = 98) and percentage of correct answers
